# Economic evaluation of stepped-care versus usual care for depression and anxiety in older adults with vision impairment: randomized controlled trial

**DOI:** 10.1186/s12888-017-1437-5

**Published:** 2017-08-01

**Authors:** Hilde P. A. van der Aa, Ger H. M. B. van Rens, Judith E. Bosmans, Hannie C. Comijs, Ruth M. A. van Nispen

**Affiliations:** 1 Department of Ophthalmology and the Amsterdam Public Health Research Institute, VU University Medical Centre, De Boelelaan 1117, 1081 Amsterdam, HV The Netherlands; 2 Department of Ophthalmology, Elkerliek Hospital, Wesselmanlaan 25, 5707 Helmond, HA The Netherlands; 3 Department of Health Sciences and the Amsterdam Public Health Research Institute, Faculty of Earth and Life Sciences, VU University Amsterdam, De Boelelaan 1105, 1081 Amsterdam, HV The Netherlands; 4 Department of Psychiatry VUmc/GGZinGeest, A.J.Ernststraat 1187, 1081 Amsterdam, HL The Netherlands

**Keywords:** Depression, Anxiety, Vision impairment, Stepped-care, Cost-effectiveness, Cost-utility, Economic evaluation

## Abstract

**Background:**

A stepped-care program was found effective in preventing depressive and anxiety disorders in older adults with vision impairment. However, before a decision can be made about implementation, the cost-effectiveness of this program should be investigated. Therefore, we aimed to compare the cost-effectiveness of stepped-care versus usual care within low vision rehabilitation.

**Methods:**

An economic evaluation from a societal perspective was performed alongside a multicenter randomized controlled trial. Data were collected by masked assessors during 24 months. Included were 265 older adults with vision impairment and subthreshold depression and/or anxiety. They were randomly assigned to stepped-care plus usual care (*n* = 131) or usual care alone (*n* = 134). Stepped-care comprised 1) watchful waiting, 2) guided self-help based on cognitive behavioral therapy, 3) problem solving treatment, and 4) referral to a general practitioner. Costs were based on direct healthcare costs and indirect non-healthcare costs. Main outcome measures were quality-adjusted life years (QALYs) and the cumulative incidence of major depressive, dysthymic and/or anxiety disorders. Secondary outcomes were symptoms of depression and anxiety.

**Results:**

Based on intention-to-treat, significant differences were found in the incidence of depressive/anxiety disorders (mean difference 0.17; 95% CI 0.06 to 0.29) and symptoms of anxiety (mean difference 1.43, 95% CI 0.10 to 2.77) in favor of stepped-care versus usual care; no significant difference was found for QALYs and symptoms of depression. Societal costs were non-significantly lower in the stepped-care group compared with the usual care group (mean difference: -€877; 95% confidence interval (CI): −8039 to 5489). Cost-effectiveness acceptability curves showed that the probability of cost-effectiveness was 95% or more at a willingness-to-pay of €33,000 per disorder prevented. The probability that stepped-care was cost-effective compared to usual care was 59% or more for a ceiling ratio of 0 €/QALY and increased to 65% at 20000 €/QALY.

**Conclusions:**

This economic evaluation shows that stepped-care is dominant to usual care, with a probability of around 60%, due to its clinical superiority and its modest cost savings. However, it depends on the willingness-to-pay of decision makers whether or not stepped-care is considered cost-effective compared with usual care.

**Trial registration:**

identifier: NTR3296, date: 13–02-2012.

**Electronic supplementary material:**

The online version of this article (doi:10.1186/s12888-017-1437-5) contains supplementary material, which is available to authorized users.

## Background

Depression and anxiety are highly prevalent in older adults with vision impairment [1, 2] and have a negative impact on overall health, vision-related disability, and quality-of-life [3–8]. Moreover, depression and anxiety generate substantial economic burden due to increased healthcare utilization and productivity losses [9–12]. Consequently, vision impairment in old age is a socio-economic problem. However, research on psychological interventions to treat and prevent mental health problems in this population is scarce [13, 14]. Moreover, apart from a few studies on the cost-effectiveness of rehabilitation and education to increase wellbeing [15, 16], economic evaluation of psychological interventions in the field of low vision is (to our knowledge) completely lacking.

Stepped-care is a model proposed to increase efficiency in mental healthcare. In stepped-care, patients receive subsequent treatment components by order of intensity, i.e. patients start with low-intensity treatments and only move on to higher-intensity treatments when sufficient response is lacking. This care model is expected to lower costs by maximizing the efficiency of resource allocation, and is, therefore, recommended by Dutch and British guidelines (e.g. the National Institute for Health and Clinical Excellence) [17–19]. Several studies outside the field of low vision have shown that stepped-care is cost-effective as compared to usual care in reducing depression and anxiety [19, 20]. Our group previously showed that stepped-care significantly reduced the incidence of depressive and anxiety disorders in older adults with vision impairment [21]. However, the cost-effectiveness of this intervention has not yet been investigated.

Therefore, the present study aimed to perform a cost-utility analysis for quality adjusted life years (QALYs) of stepped-care in comparison with usual care and to perform a cost-effectiveness analysis in preventing major depressive and anxiety disorders and reducing symptoms of depression and anxiety in older adults with vision impairment.

## Methods

### Design

An economic evaluation based on a societal perspective was performed alongside a two-armed multicenter randomized controlled trial (RCT), as described in the original protocol [22]. The trial is registered in the Dutch trial registry (identifier: NTR3296, http://www.trialregister.nl). Detailed information on the study design and intervention is provided elsewhere [21, 22].

### Participants and setting

From July 2012 to April 2013, a random sample of 3000 patients of 50 years and older from 17 outpatient clinics of three low vision rehabilitation centers in Belgium and the Netherlands were invited to participate. Of these, 30.5% (*n* = 914) provided written informed consent, and underwent baseline interviews to determine eligibility.

Patients were eligible if: a) they had subthreshold depression and/or anxiety, i.e. scored ≥16 on the Centre for Epidemiologic Studies Depression scale (CES-D) [23, 24] and/or ≥8 on the Hospital Anxiety and Depression Scale-Anxiety subscale (HADS-A) [25, 26], b) they did not meet the DSM-IV criteria of a major depressive, dysthymic and/or anxiety disorder as assessed with the Mini International Neuropsychiatric Interview (MINI) [27, 28], c) they adequately spoke the Dutch language, and d) they were not severely cognitively impaired, as assessed with the Six-item screener [29].

### Randomization

A pre-specified power calculation was based on the study of van ‘t Veer et al. [30] who evaluated the cost-effectiveness of stepped-care in the general elderly population. They found an effect size of 0.44. Based on a two-sided α ≤ 0.05, a power of 0.85, and a dropout rate of 20%, a minimum of 230 patients (115 in each arm) was needed. However, since we observed higher dropout rates than expected at the start of the RCT, more patients were included (*n* = 265).

Participants were randomly assigned to stepped-care plus usual care (*n* = 131) or usual care alone (*n* = 134). An allocation scheme was generated by a computerized random number generator stratified by outpatient clinic and based on blocks of two. After the baseline measurement, an independent researcher performed randomization, which was registered at the low vision rehabilitation center. From the beginning of September 2012 to the end of July 2015, seven telephone interviews (baseline, and after 3, 6, 9, 12, 18 and 24 months) per participant were performed at the VU University Medical Centre in Amsterdam by trained and masked research assistants. Participants were told not to divulge the nature of their treatment. Due to the nature of the stepped-care intervention, therapists and patients could not be masked.

### Intervention

The stepped-care program consisted of four steps that each lasted approximately 3 months The total intervention period lasted 12 months, followed by a 12-month follow-up.

At the end of each 3-month period, elevated levels of depression and/or anxiety (i.e. ≥16 on the CES-D and/or ≥8 on the HADS-A) induced moving on to the following step of the intervention. The first step was a period of *watchful waiting*, which involved an active decision to not directly treat the depressive and/or anxious symptoms but, instead, to intermittently re-assess these symptoms. In the second step, a *guided self-help course* based on cognitive behavioral therapy (CBT) was given. The course was available in written, digital, audio and Braille formats and was supported by trained occupational therapists. In the third step, *problem solving treatment* (PST) was offered by trained social workers and psychologists. Finally, when participants still had subthreshold depression and/or anxiety after the third step, they moved to the fourth step, which was a *referral to their general practitioner (GP)* to discuss other treatment and the use of medication. Participants who developed an actual depressive and/or anxiety disorder as assessed with the MINI, were directly referred to their GP. Usual care for both the stepped-care and usual care group included low vision rehabilitation care and/or care that was offered by other healthcare providers.

### Clinical outcomes

The primary outcomes were QALYs and the cumulative incidence of major depressive, dysthymic and/or anxiety disorders (i.e. panic disorder (without agoraphobia), agoraphobia (without a history of panic disorder), social phobia, and/or generalized anxiety disorder). The latter was assessed at every measurement time point with the Dutch MINI Plus (5.0.0) [27, 28]. QALYs were determined by measuring health-related quality-of-life at baseline and at 12 and 24 months using the EuroQol (EQ-5D-3 L) using the official telephone script; this instrument comprises five dimensions (mobility, self-care, activities of daily living, pain/discomfort and depression/anxiety) with three response options (no problems, some problems, extreme problems) [31]. The EQ-5D-3 L health states were converted to health utility scores using the Dutch tariff, in which 0 corresponds to death and 1 corresponds to full health (range − 0.33 to 1, whereby negative utilities indicate that a health state is valued as worse than death) [31]. With the area under the curve method, QALYs were calculated by multiplying the amount of time a patient spent in a particular health state by the utilities. Changes in utilities between health states were considered to be linear. Secondary outcomes were change in symptoms of depression and anxiety, as assessed with the CES-D and the HADS-A, respectively. The CES-D has 20 items and the HADS-A has 7 items evaluated on a 4-point Likert-scale [23–26]. In the cost-effectiveness analyses, the change of symptoms between the start and end of follow-up was assessed instead of the course of symptoms over time as was done in the effectiveness analysis [21]. This was done to facilitate the interpretation of the incremental cost-effectiveness ratios.

### Costs measures

Costs were collected from a societal perspective (informal care was not included). Healthcare utilization was measured using the self-rated Trimbos and iMTA questionnaire for Costs associated with Psychiatric illness (TiC-P, adapted version) [32] and valued using standard costs from the Dutch costing guideline (Table 1) [33]. Medication was valued using prices from the Royal Dutch Society for Pharmacy. Lost productivity due to absenteeism from paid and unpaid work and presenteeism were measured using the Short Form Health and Labour Questionnaire (SF-HLQ) [34]. Costs of absenteeism from paid work and presenteeism were calculated using mean age and gender-specific income values of the Dutch population and calculated according to the friction method. Compared to the human capital method which assumes any hour not worked as an hour lost, the friction method assumes that after a certain period of time (i.e. 161 days) the sick employee is replaced [35]. Thus, lost productivity costs are generated only during the friction period. Lost productivity costs from unpaid work were valued using a shadow price for informal care (€13.50/h) [33]. Both the TiC-P and SF-HLQ were administered at 6,12,18 and 24 months follow-up with a recall period of 6 months. The costs of the stepped-care program were calculated using a bottom-up approach, i.e. costs were determined by the resources each person used at each step (time, materials, etcetera). Standard costs for occupational therapists, social workers and psychologists obtained from the Dutch costing guideline were used to value these resources (see Table 1). The index year was 2013. If necessary, consumer price indices were used to correct prices [36].

**Table 1:** Unit costs to value healthcare utilization

Cost category	Unit	Unit costs (2013)^a^
General practitioner	Contact	€30.64
Company physician	Contact	€32.26
Medical specialist	Contact	€78.33
Occupational or physiotherapist	Contact	€39.16
Social worker	Contact	€70.71
Psychologist or psychiatrist in private practice	Contact	€87.03
Psychologist or psychiatrist in hospital	Contact	€186.03
Mental healthcare institute worker	Contact	€186.03
Alternative healer	Contact	€44.67
Day treatment for mental care	Day	€167.54
Admission to regular hospital	Day	€473.23
Admission to academic hospital	Day	€625.54
Admission to psychiatric hospital	Day	€252.39
Admission to rehabilitation center	Day	€369.89
Admission to nursing home	Day	€258.92
Admission to other healthcare institution^b^	Day	€497.17
Homecare	Hour	€38.07
Informal care	Hour	€13.50

^a^Valued based on standard costs from the 2009 Dutch costing guideline and indexed to the year 2013

^b^Weighted average of the unit costs for admission to a regular, academic or psychiatric hospital, a rehabilitation center or nursing home

### Statistical analyses

First, non-response analyses, dropout analyses, and comparisons of baseline differences between the stepped-care group and usual care group were performed with χ^2^-tests, independent samples t-tests, and non-parametric tests. Second, a cost-effectiveness and cost-utility analysis were performed based on the intention-to-treat principle.

The effectiveness analyses are reported elsewhere [21]. In contrast with the effectiveness analyses, in the present study missing cost and effect data were replaced using multiple imputation with chained equations (MICE) [37]. The number of imputed datasets was increased until the loss of efficiency was less than 5% resulting in 15 imputed datasets [38]. Variables in the imputation model included all outcome variables, characteristics differing between groups at baseline, variables related to missing data and variables related to the outcome variables. To account for the non-normal distribution of cost data, predictive mean matching was used in the MICE procedure [38]. Results from the multiple datasets were pooled using Rubin’s rules [39]. Bivariate regression models were used to estimate cost and effect differences, and incremental cost-effectiveness ratios (ICERs) were calculated. Bias-corrected and accelerated bootstrapping was applied to estimate 95% confidence intervals (CI) around the mean cost and effect differences (5000 replications). The bootstrapped cost-effect pairs were plotted on a cost-effectiveness (CE) plane for each outcome separately to show the uncertainty around the ICER. The net benefit approach was used to estimate a cost-effectiveness acceptability curve (CEAC) using the pooled cost and effect differences, and the pooled, bootstrapped standard errors. The CEAC shows the probability that stepped-care is cost-effective in comparison with usual care for a range of different ceiling ratios (i.e. the willingness-to-pay for one additional recovered patient), indicating decision uncertainty [40].

For the analyses, SPSS for Windows version 21 (SPSS IBM, New York, USA) and Stata/SE software, version 12 (Stata Corp LP) were used.

### Sensitivity analysis

The primary analysis was based on costs from a societal perspective using the friction method to estimate indirect non-healthcare costs.

In addition, two sensitivity analyses were performed: i) the cost-effectiveness analysis was performed from a healthcare perspective, i.e. including only direct costs, ii) also, to determine productivity losses, the human capital method was used in which every hour not worked is considered as a lost hour.

## Results

### Participant flow

Of the 3000 invited participants, responders (*n* = 914; 30%) were significantly younger than non-responders (*n* = 2086; 70%) (mean difference 4.6 years, *p* < 0.001). At 24-month follow-up, of the 265 patients eligible/willing to participate, 91 dropped-out (34.3%; 45 stepped-care and 46 usual care). Participants who dropped-out of the study more often lived in a nursing home and were significantly older than those who did not drop-out (*p* < 0.05). Common reasons for dropout were: mortality, physical or mental inability to continue, or too heavy a burden.

Table 2 presents the baseline characteristics of the two study groups: there was a significant difference in education level between the groups (*p* < 0.05).

**Table 2:** Baseline patient characteristics for the stepped-care (*n* = 131) and usual care group (*n* = 134)

Patient characteristics measured at baseline		Stepped-care (*n* = 131)	Usual care (*n* = 134)
Gender (female) (n (%))		91 (69%)	94 (70%)
Age (years) range [50–98] (mean (SD))		72.4 (12.5)	74.9 (11.9)
Education (years) range [0–16] (mean (SD))		10.4 (3.8)	9.3 (3.4)
Having work (n (%))		15 (12%)	7 (5%)
Nationality (n (%))	Dutch	116 (89%)	117 (87%)
	Belgian	14 (11%)	16 (12%)
	Other	1 (1%)	1 (1%)
Living situation (independent) (n (%))		115 (88%)	124 (93%)
Income (n (%))	Usually enough money	61 (47%)	62 (46%)
	Just enough money	55 (42%)	57 (43%)
	Not enough money	10 (8%)	15 (11%)
Cause of vision loss (n (%))	Macular degeneration	62 (47%)	60 (45%)
	Glaucoma	26 (20%)	19 (14%)
	Cataract	26 (20%)	19 (14%)
	Diabetic retinopathy	5 (4%)	4 (3%)
	Cerebral hemorrhage	5 (4%)	10 (8%)
	Other	45 (34%)	60 (45%)
Time of onset (years) range [0–79] (mean (SD))		16.0 (19.6)	14.4 (18.2)
LogMAR visual acuity (n (%))	Normal visual acuity	9 (7%)	15 (11%)
	Mild vision loss	24 (18%)	23 (17%)
	Low vision / blindness	86 (66%)	86 (64%)
Comorbidity range [0–5] (mean (SD))		1.1 (1.2)	1.2 (1.2)
History of major depressive disorder (n (%))		30 (23%)	25 (19%)
History of dysthymic disorder (n (%))		4 (3%)	1 (1%)
History of panic disorder (n (%))		8 (6%)	8 (6%)
Baseline CES-D score (mean(SD))		21.2 (6.4)	21.1 (6.7)
Baseline HADS-A score (mean(SD))		7.1 (4.1)	7.1 (3.8)
Baseline EQ-5D utility score (mean(SD))		0.7 (0.3)	0.7 (0.2)

SD standard deviation, CES-D Center for Epidemiologic Studies Depression, HADS-A Hospital Anxiety and Depression Scale-Anxiety, EQ-5D Euroqol-5 Dimensions

### Clinical outcomes

The cumulative incidence of depressive/anxiety disorders at 24-month follow-up was 0.29 in the stepped-care group and 0.46 in the usual care group. The absolute risk reduction was 0.17; this difference was significant (95% CI 0.06 to 0.29) (Table 3). In addition, imputed and pooled outcomes showed a significant difference between the stepped-care group and usual care group for the HADS-A (mean difference 1.43, 95% CI 0.10 to 2.77), and a non-significant difference for the CES-D (mean difference 2.73, 95% CI -0.28 to 5.74) and QALYs (mean difference 0.03, 95% CI -0.09 to 0.15). Note that these latter analyses are different from those performed in our earlier study.^21^

**Table 3:** Multiple-imputed effects and costs^a^ for the stepped-care (*n* = 131) and usual care group (*n* = 134) after 24 months

Outcome		Stepped-care (*n* = 131) (mean (SE))	Usual care (*n* = 134) (mean (SE))	Mean difference (95% CI)^b^
Cumulative incidence of depressive/anxiety disorders		0.29 (0.04)	0.46 (0.04)	0.17 (0.06 to 0.29)
Mean change CES-D score		6.40 (1.05)	3.67 (0.99)	2.73 (−0.28 to 5.74)
Mean change HADS-A score		1.88 (0.47)	0.45 (0.51)	1.43 (0.10 to 2.77)
QALY		1.32 (0.04)	1.28 (0.04)	0.03 (−0.09 to 0.15)
Direct healthcare costs	Medication costs	1705 (245)	1783 (419)	−78 (−938 to 505)
	Primary care	10,911 (1496)	10,124 (1631)	787 (−3754 to 4910)
	Secondary care	3783 (675)	5909 (1456)	−2126 (−5911 to 348)
	Intervention costs	262 (34)	0 (0)	262 (204 to 340)
	Total	16,661 (1691)	17,815 (2680)	−1154 (−7708 to 4328)
Indirect non healthcare costs^c^		5270 (771)	4993 (583)	277 (−1418 to 2230)
Total costs		21,931 (2035)	22,808 (2956)	−877 (−8039 to 5489)

^a^Costs are presented in € and indexed to the year 2013

^b^For cost measures bootstrapped 95% confidence intervals were used

^c^Calculated with the friction method

SE standard error, CI confidence interval, CES-D Center for Epidemiologic Studies Depression, HADS-A Hospital Anxiety and Depression Scale-Anxiety, QALY quality adjusted life year

### Costs

All participants in the stepped-care group received watchful waiting (*n* = 131), 56% received CBT-based guided self-help, 22% received PST, and 5% were referred to their GP as part of the last step of the intervention.

Cost and effect data are presented in Table 3. The mean total intervention costs amounted to €262 per participant. Although direct healthcare costs were lower for the stepped-care group compared with the usual care group, the mean difference was not significant (−€1154; 95% CI -7708 to 4328). Cost savings were mainly due to significantly lower secondary mental healthcare and hospitalization costs. Indirect non-healthcare costs based on the friction method were not significantly higher for the stepped-care group compared with the usual care group (€277; 95% CI -1418 to 2230). Total costs were lower for the stepped-care group compared to usual care (−€877; 95% CI -8039 to 5489) but the difference was not significant.

### Cost utility and cost-effectiveness

**Fig. 1:**
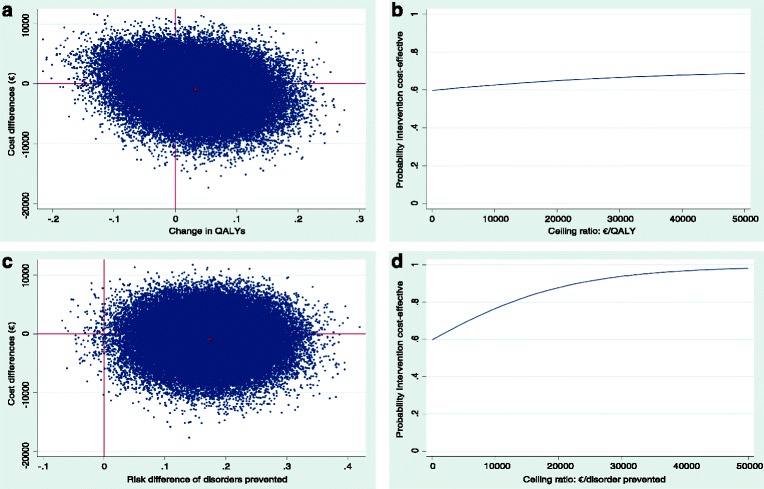
**a** Cost-effectiveness plane. Showing the change in quality adjusted life years (QALY) during 24 months follow-up in the stepped-care versus the usual care group from a societal perspective using the friction method. The red dot indicates the point estimate of the ICER, mean difference was 0.03 and €877 less costs were made in the stepped-care group. The grey dots indicate the bootstrapped cost-effects pairs reflecting the uncertainty surrounding the ICER. **b** Cost-effectiveness acceptability curve. Showing the probability that the stepped-care intervention is cost-effective compared to the control condition from a societal perspective using the friction method in change in quality adjusted life-years (QALY) over a range of values for the maximum acceptable ceiling ratio. **c** Cost-effectiveness plane. Showing the percentage of major depressive and anxiety disorders prevented during 24 months follow-up in the stepped-care versus the usual care group from a societal perspective using the friction method. The red dot indicates the point estimate of the incremental cost-effectiveness ratio (ICER, 17% of disorders were prevented and €877 less costs were made in the stepped-care group). The grey dots indicate the bootstrapped cost-effects pairs reflecting the uncertainty surrounding the ICER. **d** Cost-effectiveness acceptability curve. Showing the probability that the stepped-care intervention is cost-effective compared to the control condition from a societal perspective using the friction method in preventing major depressive and anxiety disorders over a range of values for the maximum acceptable ceiling ratio

The cost-utility analysis resulted in an ICER of −29,233 indicating that to gain 1 QALY €29,233 is saved in the stepped-care group as compared to usual care. The CE plane and the CEAC in Fig. 1 a and b show that the probability that stepped-care was cost-effective compared to usual care was 59% or more for a ceiling ratio of €0 per QALY and that this increased to 65% or more at a willingness-to-pay of €20,000 per QALY.

With regard to the cumulative incidence of depressive/anxiety disorders, the ICER was −5159 indicating that to prevent one case of depression or anxiety €5159 is saved in the stepped-care group as compared to usual care (Table 4, Fig. 1). The CE plane and the CEAC in Fig. 1 c and d show that, for a ceiling ratio of €0 per disorder prevented, the probability that stepped-care was cost-effective compared to usual care was 59%. At a willingness-to-pay of €10,000 this probability was 77%, and at a willingness-to-pay of €20,000 this probability was 88%. The probability of cost-effectiveness increased to 95% or more at a willingness-to-pay of €33,000 per disorder prevented.

**Table 4:** Results of the cost-effectiveness and cost-utility analyses

Outcome	Analysis	Mean cost difference (stepped-care – usual care (bootstrapped 95% CI))	Mean change difference (stepped-care – usual care (95% CI))	ICER	CE-plane^a^			
					NE	SE	SW	NW
Disorder	Societal perspective (friction)	−877 (−8039 to 5489)	0.17 (0.06 to 0.29)	−5159	41%	59%	0%	0%
	Societal perspective (human capital)	200 (−7035 to 6829)		1176	53%	47%	0%	0%
	Healthcare perspective	−1154 (−7708 to 4328)		−6788	37%	63%	0%	0%
CES-D	Societal perspective (friction)	−877 (−8039 to 5489)	2.73 (−0.28 to 5.74)	−321	39%	58%	2%	1%
	Societal perspective (human capital)	200 (−7035 to 6829)		73	51%	46%	1%	2%
	Healthcare perspective	−1154 (−7708 to 4328)		−423	35%	62%	2%	1%
HADS-A	Societal perspective (friction))	−877 (−8039 to 5489)	1.43 (0.10 to 2.77)	−613	40%	58%	2%	0%
	Societal perspective (human capital	200 (−7035 to 6829)		140	52%	46%	1%	1%
	Healthcare perspective	−1154 (−7708 to 4328)		−807	36%	63%	1%	0%
QALY	Societal perspective (friction)	−877 (−8039 to 5489)	0.03 (−0.09 to 0.15)	−29,233	25%	45%	14%	16%
	Societal perspective (human capital)	200 (−7035 to 6829)		66,667	34%	36%	11%	19%
	Healthcare perspective	−1154 (−7708 to 4328)		−38,467	23%	48%	16%	13%

Cost-effectiveness and cost-utility analyses were based on: 1) societal perspective and the friction method, 2) societal perspective and the human capital method, and 3) healthcare perspective. CI confidence interval, CES-D Center for Epidemiologic Studies Depression, HADS-A Hospital Anxiety and Depression Scale-Anxiety, QALY quality adjusted life year, ICER incremental cost-effectiveness ratio, CE cost-effectiveness, NE north-east quadrant, SE south-east quadrant, SW south-west quadrant, NW north-west quadrant. ^a^Effect estimates for Disorder, CES-D and HADS-A were multiplied by −1 in the CE plane to maintain the usual meaning of the quadrants

For the CES-D and the HADS-A, cost-effectiveness acceptability curves show that the probability of cost-effectiveness was 60% for both outcomes for a ceiling ratio of €0 per point improvement on the CES-D/HADS-A; this probability increased to 95% or more at a willingness-to-pay of €2500 per point improvement on the CES-D and €4000 per point improvement on the HADS-A (Table 4).

### Sensitivity analysis

Based on the societal perspective with the human capital method (non-significant) higher total costs were found for the stepped-care group compared to the usual care group (mean difference 200, 95% CI -7035 to 6829) (Table 4). The cost-effectiveness acceptability curves show that the probability of cost-effectiveness was 47% or more for a ceiling ratio of €0 per disorder prevented and that this increased to 95% or more at a willingness-to-pay of €42,500 per disorder prevented. Based on the healthcare perspective, compared with the main analysis a larger cost difference was found, with stepped-care being less costly than usual care (mean difference − 1154, 95% CI -7708 to 4328) (Table 4). The cost-effectiveness acceptability curves show that the probability of cost-effectiveness was 59% or more for a ceiling ratio of €0 per disorder prevented and that this increased to 95% or more at a willingness-to-pay of €26,000 per disorder prevented (see Additional file 1: Figure S1 with CE-planes and CEACs).

## Discussion

This economic evaluation shows that stepped-care is dominant to usual care, with a probability of around 60%, due to its clinical superiority and its modest (non-significant) cost savings, mainly due to lower secondary mental healthcare use and hospitalization, as compared to usual care. Mean intervention costs are low (i.e. €262 per participant) and comparable to other stepped-care programs [20, 41]. Based on the cost-effectiveness acceptability curves, it is shown that the probability that the intervention is cost-effective compared to usual care is 95% or more when society is willing to pay €33,000 per disorder prevented. Lost productivity costs were higher for stepped-care compared to usual care. However, because in this older population only a few participants had a paid job (8%), the analysis was also performed from a healthcare perspective (including only direct healthcare costs); this showed slightly more positive outcomes. Based on this perspective there is a 95% probability of stepped-care being cost-effective compared to usual care when decision makers are willing to pay €26,000 per disorder prevented. Although lower than in the main analysis, this amount is still relatively large and decision makers have to decide whether this is acceptable.

Although stepped-care was significantly more effective in preventing depressive and anxiety disorders compared with usual care, the difference in QALYs, in favor of stepped-care, was not statistically significant. QALY is a measure of health-related quality-of-life (measured with the EQ-5D in this study), including various dimensions of which mental health is covered in only one dimension. When analyzing the dimensions separately, stepped-care significantly reduced problems with activities of daily living and depression/anxiety compared with usual care; however, no significant difference was found on the other dimensions (i.e., mobility, self-care, pain/discomfort) (data not shown). The lack of an overall significant effect might be explained by the nature of the outcome, i.e. because stepped-care was specifically aimed at improving mental health, similar improvements in overall quality-of-life cannot be expected. In addition, the impact of vision loss itself on different elements of health-related quality of life is likely to influence overall quality of life in this specific population, but these effects cannot be separated. Moreover, the EQ-5D with three-level response options has limited ability to distinguish small to moderate differences in health status [42].

### Strengths and limitations

A strength of this study is the pragmatic design that was chosen. This helps policymakers make evidence-based decisions on whether implementation of stepped-care can be considered an efficient allocation of scarce resources in real-life situations and to increase generalizability of the results. Second, a long follow-up period was chosen to assess long-term treatment effects. Third, treatment arms appeared to be well randomized with no relevant differences in baseline characteristics between the stepped-care and usual care group. A limitation of this study is the high dropout rate: 34% in total. However, there was no significant difference in dropout between the stepped-care and usual care group and there was sufficient statistical power. Missing data were dealt with by applying multiple imputation techniques, which is the preferred method in economic evaluations [43]. Second, the relatively large recall periods for healthcare utilization and work productivity (6 months) may have introduced recall bias. Third, informal care was not included in the total costs from a societal perspective. However, it is hard to estimate how inclusion of informal care costs would have influenced our societal cost estimates. Fourth, although the pragmatic design of our study increases generalizability and the outcomes could be used for stepped-care in different settings, strictly speaking our outcomes can only be generalized to visually impaired older adults who are registered at a low vision rehabilitation organization.

### Implications for practice and future research

This study shows that stepped-care is dominant to usual care (with a probability of around 60%) in treating mental health problems in visually impaired older adults. Stepped-care enables professionals to efficiently deploy their limited resources by initially offering low intensity and low cost interventions and only moving on to higher-intensity and more costly interventions when sufficient response is lacking. The stepped-care program is effective in preventing major depressive/anxiety disorders as compared to usual care; the probability that stepped-care is cost-effective compared to usual care is 95% or more at a willingness-to-pay of €33,000 per disorder prevented. Future studies should investigate how the cost-effectiveness of stepped-care can be improved. Possible options for this are: offering interventions tailored to personal needs and symptom severity, e.g. varying the watchful waiting period [44], and directly offering higher intensity interventions to persons with a history of depressive/anxiety disorder [21]. In addition, other evidence-based treatment options (e.g. exercise programs, e-mental health) could be added to the model. Moreover, the present study, which investigates both the clinical effectiveness and cost-effectiveness of a promising intervention, could serve as an example for other intervention studies in the field of low vision. Considering that vision impairment is ubiquitous in an aging population and that resources in healthcare are scarce, such economic evaluations are highly relevant.

## Additional file

Additional file 1: Figure S1. Cost-effectiveness planes and cost-efectiveness acceptability curves for the sensitivity analyses. (DOCX 56 kb)

## Abbreviations

CBT: Cognitive behavioral therapy
CE: Cost-effectiveness
CEAC: Cost-effectiveness acceptability curve
CES-D: Centre for Epidemiologic Studies Depression scale
CI: Confidence interval
EQ-5D: EuroQol 5-Dimensions
GP: General practitioner
HADS-A: Hospital Anxiety Depression Scale – Anxiety
ICER: Incremental cost-effectiveness ratios
iMTA: Questionnaire for Costs associated with Psychiatric illness
MICE: Multiple imputation techniques with chained equations
MINI: Mini International Neuropsychiatric Interview
PST: problem solving treatment
QALY: Quality adjusted life year
RCT: Randomized controlled trial
SF-HLQ: Short Form Health and Labor Questionnaire
TiC-P: Trimbos
