# Supplementary figures and images for: Economic evaluation of stepped-care versus usual care for depression and anxiety in older adults with vision impairment: randomized controlled trial
Source: BMC Psychiatry. 2017 Aug 1;17:280. doi: 10.1186/s12888-017-1437-5 (PMC5539614; doi:10.1186/s12888-017-1437-5)

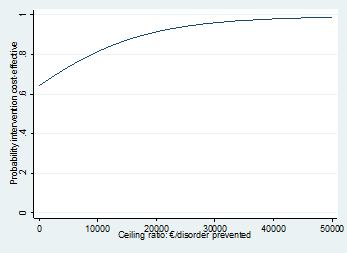

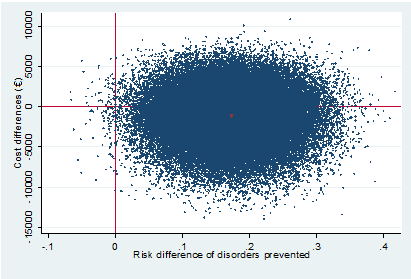


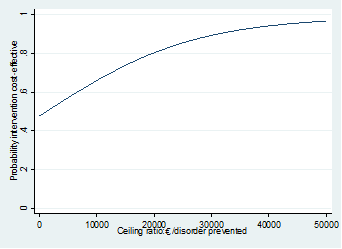

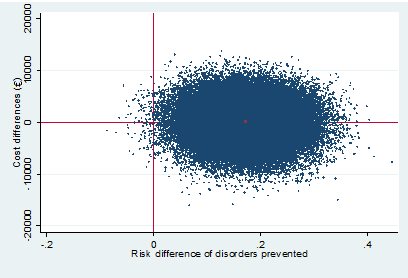

Supplement: Additional file 1: Figure S1. — Cost-effectiveness planes and cost-efectiveness acceptability curves for the sensitivity analyses. (DOCX 56 kb) [file 12888_2017_1437_MOESM1_ESM.docx]
